# Large Language Models Could Revolutionize Health Care, but Technical Hurdles May Limit Their Applications

**DOI:** 10.2196/71618

**Published:** 2025-06-25

**Authors:** Diva Beltramin, Cédric Bousquet, Théophile Tiffet

**Affiliations:** 1 Medical Information Department, Hospices Civils de Lyon, 3 Quai des Célestins, Lyon, 69002, France, 33 72357066; 2 Laboratory of Medical Informatics and Knowledge Engineering in e-Health, Sorbonne University, Paris, France; 3 Public Health and Medical Information Unit, Saint-Étienne University Hospital Center, Saint-Étienne, France; 4 Laboratoire Inserm Santé Ingénierie Biologie, U1059, Dysfonction Vasculaire et Hémostase, Université Jean Monnet, Saint-Étienne, France

**Keywords:** large language model, LLMs, digital health, medical diagnosis, treatment, multimodal data integration, technological fairness, artificial intelligence, AI, natural language processing, NLP

Zhang et al [1] recently published an article in the *Journal of Medical Internet Research* titled “Revolutionizing Health Care: The Transformative Impact of Large Language Models in Medicine.” The authors synthesized all the possible applications of large language models (LLMs) very well, not only detailing applications related to clinical medicine, but also offering some examples of LLMs’ potential in a broader hospital environment and in public health policies. It was not the authors’ objective in their Viewpoint paper to explain how these applications would be implemented, but we believe that the next steps in their research should also consider the technical hurdles of implementing LLM applications. We also observed a few minor inaccuracies in the way the authors distinguished encoder models like bidirectional encoder representations from transformers (BERT) and decoder models like generative pretrained transformers (GPTs).

The authors reproduced a figure (“The Transformer – model architecture”; the first figure in their paper) from the famous 2017 paper “Attention Is All You Need” by Vaswani et al [2] (the original figure was captioned “The architectural designs of LLMs”). However, they represented a nonexistent connection of layers between BERT and GPT models. Encoder models like BERT use encoding-only blocks, while GPT models use decoder-only blocks. Therefore, there is no encoder/decoder attention layer in the GPT model.

Moreover, while there is still a lack of evidence for the use in medicine of LLMs that take only text as input, there is even less evidence for the use of multimodal LLMs. Of course, LLMs can easily adapt to any kind of image and can produce a coherent medical report. However, in highly specialized fields such as computed tomography scans, magnetic resonance imaging, or digital histopathology [3], fine-tuned deep learning models could have better performance in image interpretation. LLMs are not necessarily a medical Swiss Army knife, and we should not force their use everywhere, as other technologies exist that are more performant on specific tasks.

Another example is in the authors’ third figure (“Integration of LLMs in health care systems across different scales”), in which the authors suggest that LLMs should be used to perform resource allocation, even though such resources are not based on unstructured text data but on structured data implying tasks, actors, and duration of interventions. Already existing techniques such as operational research rely on mathematical approaches that help to identify the optimum corresponding to the highest-performing organization. We believe that the authors should evaluate the technical solutions already available before proposing applications based only on LLMs.

Technical details should include the resolution of problems related to interoperability between the electronic health record and LLMs, given that it is necessary that LLMs can access patient data. Expert systems, such as DXplain [4] or Internist-1 [5], that help clinicians in the diagnostic process already exist, but despite having high performance, they were discarded because patient data had to be entered into the expert system.

To conclude, we encourage the authors in their approach and recommend they dive into more technical details in the implementation of LLM-based applications.

## Abbreviations

BERT: bidirectional encoder representations from transformers
GPT: generative pretrained transformer
LLM: large language model
